# Reduced dopamine function within the medial shell of the nucleus accumbens enhances latent inhibition

**DOI:** 10.1016/j.pbb.2010.11.025

**Published:** 2011-03

**Authors:** A.J.D. Nelson, K.E. Thur, R.R. Horsley, C. Spicer, C.A. Marsden, H.J. Cassaday

**Affiliations:** Institute of Neuroscience, School of Psychology, University of Nottingham, United Kingdom; Institute of Neuroscience, School of Biomedical Sciences, University of Nottingham, United Kingdom

**Keywords:** Latent inhibition, Nucleus accumbens, Core, Shell, Dopamine

## Abstract

Latent inhibition (LI) manifests as poorer conditioning to a CS that has previously been presented without consequence. There is some evidence that LI can be potentiated by reduced mesoaccumbal dopamine (DA) function but the locus within the nucleus accumbens of this effect is as yet not firmly established. Experiment 1 tested whether 6-hydroxydopamine (6-OHDA)-induced lesions of DA terminals within the core and medial shell subregions of the nucleus accumbens (NAc) would enhance LI under conditions that normally disrupt LI in controls (weak pre-exposure). LI was measured in a thirst motivated conditioned emotional response procedure with 10 pre-exposures (to a noise CS) and 2 conditioning trials. The vehicle-injected and core-lesioned animals did not show LI and conditioned to the pre-exposed CS at comparable levels to the non-pre-exposed controls. 6-OHDA lesions to the medial shell, however, produced potentiation of LI, demonstrated across two extinction tests. In a subsequent experiment, haloperidol microinjected into the medial shell prior to conditioning similarly enhanced LI. These results underscore the dissociable roles of core and shell subregions of the NAc in mediating the expression of LI and indicate that reduced DA function within the medial shell leads to enhanced LI.

## Research Highlights

► 6-OHDA depletion of DA in nucleus accumbens enhances latent inhibition. ► Haloperidol-induced blockade of DA in nucleus accumbens enhances latent inhibition. ► Shell not core subregion mediates this effect of reduced accumbal DA function.

## 1 Introduction

Latent inhibition (LI) refers to the process whereby non-reinforced pre-exposure to a stimulus normally reduces the level of associative learning that the pre-exposed stimulus can support (Lubow and Moore, 1959). LI is disrupted by amphetamine in both rats (Solomon et al., 1981; Weiner et al., 1984) and humans (Gray et al., 1992; Kumari et al., 1999) and is absent in acutely ill schizophrenia patients (Baruch et al., 1988). Conversely, LI is potentiated by antipsychotics in both rats (Weiner and Feldon, 1987; Shadach et al., 2000) and humans (Williams et al., 1997). Consequently, LI has received considerable attention as a putative animal model of cognitive deficits in schizophrenia (e.g., Weiner, 1990, 2003; Gray et al., 1991; Weiner and Arad, 2009).

Investigations into the neural substrates of LI have shown that the nucleus accumbens (NAc) and its afferent connections from the entorhinal cortex, hippocampus and basalateral amygdala play a key role in the regulation of LI (for review see Weiner, 2003). The demonstration that LI is acutely sensitive to manipulations of forebrain dopamine (DA) has led to a particular focus on the contribution of the NAc and its dopaminergic innervation to mediating the expression of LI. For example, infusions of amphetamine into the NAc have been shown under some circumstances to abolish LI (Solomon and Staton, 1982; Joseph et al., 2000; cf. Killcross and Robbins, 1983; Ellenbroek et al., 1997; Nelson et al., in press) and the disruptive effects of systemic amphetamine on LI can be blocked by intra-accumbal infusions of the DA antagonist haloperidol (Joseph et al., 2000). Similarly, 6-OHDA-induced DA depletion of the NAc leads to the emergence of LI under conditions that do not produce LI in control animals (Joseph et al., 2000).

However, it is now indisputably established that the NAc is a heterogeneous structure with at least two morphologically and neurochemically distinct subregions, shell and core (Zahm and Brog, 1992), that have also been functionally dissociated (e.g., Parkinson et al., 1999; Di Chiara, 2002; Sellings and Clarke, 2003; Floresco et al., 2006). Consistent with these reports, there is very good evidence that the core and shell subterritories of the NAc are differentially involved in LI. Excitotoxic and electrolytic lesions to the shell disrupt LI but the effect is spared by similar lesions to core NAc (Tai et al., 1995; Weiner et al., 1996; Gal et al., 2005; Pothuizen et al., 2005). These findings have led to the suggestion that the core mediates responding to CS-reinforcement contingencies at conditioning and the shell inhibits this core-based switching mechanism (Weiner, 1990, 2003; Gal et al., 2005; Weiner and Arad, 2009).

To date, the effects of intra-accumbal manipulations of DA to differentiate the role of the core and shell subregions on LI have not been reported. Furthermore, earlier investigations into the role of DA in NAc sub-regions in LI have used experimental parameters designed to produce reliable LI in the vehicle controls (Nelson et al., in press) — because these are the parameters suitable to test for disruption — but few studies have examined the role of NAc subregions under experimental conditions that do not yield LI in controls (Joseph et al., 2000). Experiment 1 tested the effects of 6-OHDA lesions targeted at the core and medial shell on LI using experimental parameters specifically designed to prevent the emergence of LI in normal rats (weak pre-exposure). This was followed in Experiment 2, by an examination of the effect of haloperidol microinjected prior to conditioning into the core or medial shell using the same behavioral parameters as Experiment 1.

## 2 Materials and methods

### 2.1 Subjects

In each experiment, the subjects were 72 adult male Wistar rats (Charles River, UK), caged in pairs on a 12:12 h light/dark cycle with food and water *ad libitum*. Rats were handled for approximately 10 min per day for 1 week and then at mean weight 234 g (range 198–270 g) underwent surgery. In Experiment 1, 24 rats were randomly allocated to each of the core and shell lesion group and a total of 24 rats were allocated to the lesion control group (12 rats were vehicle-injected at the core coordinates and 12 rats were vehicle-injected at the shell coordinates). In Experiment 2, 34 animals were successfully implanted with guide cannulae aimed at the core and 34 with guide cannulae aimed at the shell.

All procedures were carried out in accordance with the United Kingdom (UK) Animals Scientific Procedures Act 1986, Project Licence number: PPL 40/3163. The UK Act ensures full compliance with the “Principles of laboratory animal care” (NIH publication no. 86-23, revised 1985) and the EC Directive 86/609/EEC for animal experiments.

### 2.2 Behavioral apparatus

Six identical fully automated conditioning chambers, housed within sound-attenuating cases containing ventilation fans (Cambridge Cognition, Cambridge, UK), were used. Each of the inner conditioning chambers consisted of a plain steel box (25 cm × 25 cm × 22 cm high) with a Plexiglas door (27 cm × 21 cm high) at the front. The floor was a shock grid with steel bars 1 cm apart and 1 cm above the lip of a 7 cm deep sawdust tray. A waterspout was mounted on one wall. The spout was 5 cm above the floor and connected to a lickometer supplied by a pump. Licks were registered by a break in the photo beam within the spout, which also triggered water delivery of 0.05 ml per lick. The waterspout was illuminated when water was available. A loudspeaker was set in the roof and delivered a 5 s mixed frequency noise set at 85 dB (including background) as the conditioned stimulus (CS). Footshock of 1 s duration and 1 mA intensity provided the unconditioned stimulus (UCS). This was delivered through the grid floor by a constant current shock generator (pulsed voltage: output square wave 10 ms on, 80 ms off, 370 V peak under no load conditions, MISAC Systems, Newbury, UK). Stimulus control and data collection was by an Acorn Archimedes RISC computer programmed in Basic with additional interfacing using an Arachnid extension (Cambridge Cognition).

### 2.3 Behavioral procedure

Water deprivation was introduced 1 day prior to shaping. Thereafter, the animals received 1 h and 15 min of *ad libitum* access to water in their home cage in addition to water in the experimental chambers.

#### 2.3.1 Pre-conditioning

Rats were shaped for 1 day until all drank from the waterspout and were then individually assigned to a conditioning box. They then drank in the allocated box for 15 min (timed from first lick) on each of 5 days. The drinking spout was illuminated throughout, but no other stimuli were presented. Latency to first lick was measured as an indicator of habituation to the experimental context. Total number of licks was also recorded each day to assess any pre-existing differences in drinking (prior to conditioning).

#### 2.3.2 Pre-exposure

The pre-exposed (PE) animals received 10 5 s CS presentations with an average inter-stimulus-interval of 60 s. The non-pre-exposed (NPE) control animals were confined to the boxes for an identical period of time (10 min) without receiving the CS presentations. Water was not available and the waterspout was not illuminated during the pre-exposure session.

#### 2.3.3 Conditioning

Conditioning was conducted on the following day. No water was available and the waterspout was not illuminated. There were 2 conditioning trials in which the UCS footshock was delivered following termination of the CS. The first pairing of CS and UCS was presented after 5 min had elapsed, and the second pairing was 5 min after the first, followed by a further 5 min left in the apparatus. In the absence of drinking, there were no behavioral measures to record.

#### 2.3.4 Reshaping

24 h after conditioning, animals were reshaped following the same procedure as in pre-conditioning sessions. This was in order to re-establish drinking after conditioning. Reshaping also provided measures of conditioning to the box context (latency to first lick).

#### 2.3.5 Extinction tests

On the day following reshaping, water was available throughout the test and the waterspout was illuminated. The program was triggered by each rat's first lick. Then once the rat had made 50 licks, the CS was presented for 15 min. Excluding the time to first lick, the latency to make 50 licks in the absence of the CS (the A period) provided a measure of any individual variation in baseline lick responding. This was compared with the time taken to complete 50 licks following CS onset (B period) in a suppression ratio (A / (A + B)) to assess the level of conditioning to the CS, adjusted for any individual variation in drink rate. On the next day, animals underwent an identical extinction test (Experiment 1 only).

### 2.4 Experiment 1

#### 2.4.1 Surgery

This took place prior to any behavioral procedures. All rats were pre-treated with desipramine (20 mg/kg, s.c.) 40 min prior to surgery. Anaesthesia was induced by isoflurane (4%) in a N_2_O/O_2_ (1:2, v/v) mixture and maintained with isoflurane (1–2%). Stereotaxic surgery was conducted with the incisor bar set at − 3.3 mm below the intra-aural line. A craniotomy was performed with a 1 mm hand drill and the dura was cut to expose the cortex. Rats received bilateral infusions of 6-OHDA or vehicle into either NAc core or medial shell at the following stereotaxic coordinates from bregma — core: AP + 1.6 mm, ML ± 1.8 mm, DV − 6.8 mm; medial shell: AP + 1.3 mm, ML ± 0.8 mm, DV − 6.4 mm and 7.0 mm (Paxinos and Watson, 2005). DV coordinates were taken from dura (1 infusion at each DV coordinate). Infusions were made via a 31 gauge stainless steel injector attached by polythene tubing to a 1 μl Hamilton syringe. 6-OHDA hydrobromide (24 mg/ml as salt dissolved in vehicle; Sigma, UK) or vehicle (0.9% saline/ascorbic acid 0.01% w/v) was infused manually over 2 min on each side in a volume of 0.5 μl (core) or as 2 infusions of 0.25 μl (medial shell). The injectors were left *in situ* for 5 min to allow absorption of the bolus and to minimize spread of the toxin.

Rimadyl (0.03 ml s.c.) provided post-operative analgesia. Animals were allowed 5 days recovery before the commencement of behavioral testing.

#### 2.4.2 Neurotransmitter assay

Following the completion of behavioral testing, the animals were humanely killed by dislocation of the neck and then decapitated. The brains were removed and dissected on a cold tray using a brain matrix (Harvard Instruments, USA). The brain samples were then frozen on dry ice and stored at − 80 °C. A stainless steel micropunch was used to remove samples of tissue from the following (left and right) brain regions: core NAc, medial shell NAc, dorsolateral striatum (DLS), prelimbic (PL) cortex and infralimbic (IL) cortex. The tissue samples were homogenised in 0.1 M PCA solution and centrifuged. Neurotransmitter levels in the samples were determined by high-pressure liquid chromatography (HPLC) with electrochemical detection. Bradford assay was used to adjust for protein content.

### 2.5 Experiment 2

#### 2.5.1 Surgery

Animals underwent the same surgical procedure as in Experiment 1, except that bilateral stainless steel guide cannulae (22 gauge, length 11 mm below guide; Plastic One, Roanoke, VA, USA) were implanted to allow subsequent micro-injection (see 2.5.2). Guide cannulae were aimed at the NAc core or medial shell at the following stereotaxic coordinates from bregma — core: AP + 1.6 mm, ML ± 1.9 mm, DV − 4.8 mm; medial shell: AP + 1.3 mm, ML ± 0.75 mm, DV − 4.7 mm. Cannulae were held in place by dental cement and anchored to the skull with four fixing screws located on different bone plates. Removable obturators were inserted into the guide cannulae to prevent the cannulae from blocking.

As in Experiment 1, Rimadyl (0.03 ml s.c.) provided post-operative analgesia. Animals were allowed 5 days recovery before the commencement of behavioral testing.

#### 2.5.2 Haloperidol microinjection

Haloperidol (Antigen Pharmaceuticals, Croydon, UK) was dissolved in saline and the pH was adjusted to ~ 6.5 with NaOH. It was injected at a dose of 0.5 μg/0.5 μl per hemisphere prior to the conditioning stage of Experiment 2 (Joseph et al., 2000). Rats were lightly restrained, the dust caps and obturators were removed, and 31 gauge stainless steel infusion cannulae that protruded 2 mm beyond the tip of the guide cannulae were inserted into either the core or shell of the NAc. The infusion cannulae were connected to two 5 μl syringes mounted on an infusion pump. Haloperidol was injected over 1 min and the infusion cannulae were left in situ for a further 1 min to allow absorption of the bolus. The infusion cannulae were then removed and the obturators and dust caps replaced. The animals were returned to the home cage before the onset of conditioning which took place 10 min after completion of the microinjection. Control animals underwent the identical procedure but received infusions of saline. In total, 22 animals received microinjections of haloperidol into the core and 24 into the shell; 11 animals were infused with saline into the core and 11 into the shell (see 3.2.1 for final cell sizes).

#### 2.5.3 Histological assessment of cannulae placement

Following the completion of behavioral testing, rats received a lethal dose of sodium pentobarbitone. To aid verification of the placement of the cannulae tips, infusion cannulae were inserted and 0.5 μl Pontamine sky blue dye was infused following the microinfusion procedure described above. Then the animals were immediately decapitated with a guillotine. The headcaps and guide cannulae were removed and the brain taken out and fixed in 4% formal saline for at least 7 days. Slices (80 μm thick) were made using a vibratome and were mounted onto gelatine-coated slides. Placement of the infusion cannulae tips was verified with a light microscope and the atlas of Paxinos and Watson (2005).

### 2.6 Design and analysis

**Fig. 1:**
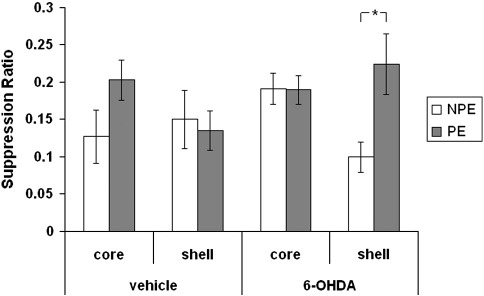
Mean suppression ratio (± S.E.M.) to the CS for non-pre-exposed (NPE: white bars) and pre-exposed (PE: light grey) groups following vehicle or 6-OHDA lesions to either the core or shell subregions of the nucleus accumbens.

**Fig. 2:**
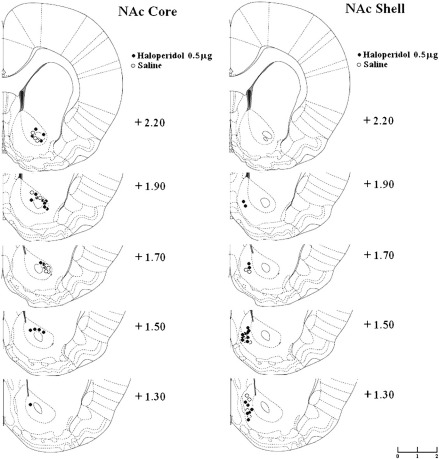
Histological assessment of cannula placements within the nucleus accumbens. Representative coronal sections from rats that received microinjections of 0.5 μg haloperidol or saline into the NAc core and NAc shell. Outlines are reproduced from Paxinos and Watson (2005) and coordinates refer to the distance in millimetres anterior to bregma.

Initial analyses of the behavioral results revealed unexpected effects of the vehicle infusions into the NAc subregions on the behavioral procedures under investigation (see Figs. 1 and 2). This difference in the baseline level of LI would be occluded by the use of collapsed controls and hence the data are presented for the individual vehicle-injected groups. As the aim of the current experiments was to identify the neuroanatomical locus within the NAc of the known effects of 6-OHDA and haloperidol on latent inhibition, the critical comparisons at test are the effects of CS pre-exposure in each NAc subregion following the 6-OHDA (Experiment 1) and haloperidol (Experiment 2) treatments. Thus, in order to allow focused comparisons (Abelson, 1995) and in view of the unequal cell sizes the behavioral results are analyzed separately by treatment for each Experiment. For each treatment, (6-OHDA or vehicle in Experiment 1, haloperidol or saline in Experiment 2), there are 4 experimental groups run in separate 2 × 2 analyses of variance (ANOVA) of matched cell size with between subject factors: pre-exposure (PE or NPE) and subregion (core or shell). In addition, the baseline lick data are analyzed with day (at 5 levels) as a within subject factor. T-tests were used for planned comparisons. In line with earlier work, the animals underwent 2 extinction tests in Experiment 1 (Joseph et al., 2000). Thus the repeated measures ANOVAs to check for differences across the tests had an additional within subjects factor with 2 levels (test 1 and test 2).

## 3 Results

### 3.1 Experiment 1

#### 3.1.1 Neurotransmitter assay

Quantification of the lesions by HPLC revealed that 6 animals showed no evidence of NAc DA depletion, consequently these animals were not considered for further analysis.

Tables 1a and 1b display the levels (pmol/μg protein) of DA and noradrenalin (NA) in the 5 brain regions from which samples were taken as (A) absolute levels and (B) as the percentage depletion relative to vehicle-injected levels. As is clear from the tables, infusion of 6-OHDA into core produced a mean depletion in DA of 64% in the target structure and 47% in the adjacent shell region compared to vehicle-injected levels. Similarly, the shell lesion led to greater depletion in the shell sample (68%) compared to the DA depletion seen in the adjacent core (44%). Significantly, following both infusions, the depletions found in the adjacent region were lower than the depletion produced by the lesion targeted on that region. Some changes in DA levels were also found in the DLS and PL samples following infusion of 6-OHDA into the core. The desipramine pretreatment did not fully protect NA in that there were effects of the 6-OHDA core infusion on NA in various of the brain regions sampled. However, the pattern of NA depletion did not match that of the DA depletion.

#### 3.1.2 Behavioral

##### 3.1.2.1 Pre-conditioning

All animals drank in the chambers and in both 2 × 2 analyses there was a gradual increase in total licks across the 5 days of lick training (min F_(4,152)_ = 2.93, p < 0.05). This increase over days was unaffected by subregion as there were no overall effects of this factor (max F_(1,40)_ = 2.38, p = 0.13). Similarly, in both 2 × 2 analyses, the latency to first lick declined over the 5 days of pre-training (min F_(4,84)_ = 9.3, p < 0.001). This increasing readiness to drink was unaffected by subregion in the 6-OHDA treated animals (F < 1) but there was an effect of subregion in the vehicle-infused animals in that the latencies in the shell-vehicle group declined more slowly over the 5 days of pre-conditioning relative to the core-vehicle group (F_(1,21)_ 11.44, p < 0.01).

##### 3.1.2.2 Reshaping

There was no effect of any of the experimental factors on the log (10) times (s) to complete the first lick in the reshape session (max F_(1,38)_ = 2.12, p = 0.15). Similarly, there were no differences between the groups in the total amount drunk during the reshape session (max F_(1,38)_ = 1.14, p = 0.29).

##### 3.1.2.3 Extinction tests

None of the experimental groups differed in the time to make 50 licks in the absence of the tone (A period) in either extinction test (max F_(1,38)_ = 1.73 p = 0.19). Analysis of the 2 tests revealed an effect of test in both vehicle (F_(1,19)_ = 44.25, p < 0.001) and 6-OHDA groups (F_(1,38)_ = 47.23, p < 0.001) as the overall level of conditioned suppression declined across the 2 tests consistent with extinction to the CS. However, this factor did not interact with either subregion or pre-exposure (max F_(1,38)_ = 3.03, p = 0.09) and hence the data are presented in Fig. 1 collapsed across the 2 tests.

Inspection of Fig. 1 reveals that as expected with limited CS pre-exposure there was no evidence of an effect of pre-exposure on conditioning to the CS in the vehicle-injected groups. ANOVA yielded no effect of subregion, pre-exposure or interaction between these factors (max F_(1,19)_ = 1.95, p = 0.18) in the vehicle-treated groups. Similarly, there was no effect of pre-exposure on conditioning to the CS in the 6-OHDA-core-lesioned group. However, Fig. 1 shows that the 6-OHDA-shell lesion appeared to potentiate LI as the shell PE group showed markedly less suppression to the CS than the NPE group. This description of the data was supported statistically by an effect of pre-exposure (F_(1,38)_ = 5.27, p < 0.05) but also critically a pre-exposure × lesion interaction (F_(1,28)_ = 5.45, p < 0.05). This interaction arose because there was no difference between the PE and NPE animals in the 6-OHDA-core group (t < 1) but a robust LI effect in the 6-OHDA-shell group (t_(21)_ = 2.92, p < 0.01).

### 3.2 Experiment 2

#### 3.2.1 Histological assessment

Histological assessment confirmed that 10 animals had misplaced cannulae (7 in haloperidol-injected groups, 3 in saline-injected groups). The remaining placements were successfully positioned in shell versus core NAc (Fig. 2; cf. Nelson et al., in press). The final sample sizes were as follows: 20 haloperidol-core; 19 haloperidol-shell; 10 saline-core; 9 saline-shell.

#### 3.2.2 Behavioral

##### 3.2.2.1 Pre-conditioning

There was a gradual increase in the amount drunk across the 5 days of pre-conditioning (min F_(4,148)_ = 2.92, p < 0.05) but there was no effect of subregion (all Fs < 1). Conversely, there was a decrease in the latency to first lick (min F_(4,68)_ = 3.51, p < 0.05). This decrease was unaffected by subregion in the saline group (F < 1). In the haloperidol group, the shell group had overall higher latencies than the core group (F_(1,37)_ = 4.17, p < 0.05) but by day 5 there were no differences between the two subregions (F < 1).

##### 3.2.2.2 Reshaping

There was no effect of any of the experimental factors on the log (10) times (s) to complete the first lick in the reshape session (max F_(1,15)_ = 3.12, p = 0.1). Similarly, there were no differences between the groups in the total amount drunk during the reshape session (all Fs < 1).

##### 3.2.2.3 Extinction test

Analysis of the A period (time to make 50 licks in the absence of the CS) revealed no effect of pre-exposure or subregion in either saline or haloperidol-infused animals (all Fs < 1).

**Fig. 3:**
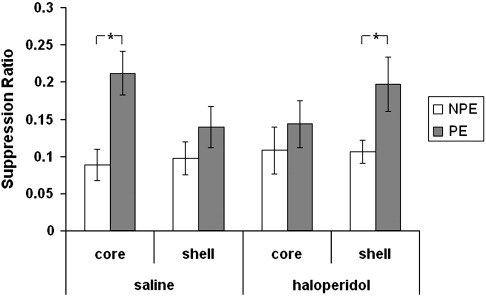
Mean suppression ratio (± S.E.M.) to the CS for non-pre-exposed (NPE: white bars) and pre-exposed (PE: light grey) groups following saline or haloperidol injections in either the core or shell subregions of the nucleus accumbens.

The mean suppression ratios to the CS in the extinction test are presented in Fig. 3. ANOVA revealed an effect of pre-exposure in both saline- (F_(1,15)_ = 10.78, p < 0.005) and haloperidol-injected animals (F_(1,35)_ = 4.24, p < 0.05) but no interaction by subregion (max F_(1,15)_ 2.59, p = 0.13). Nevertheless, as is clear from Fig. 3 the level of conditioning to the CS was not equivalent across the subregions and infusion groups. Enhanced LI was demonstrated as reduced suppression in the pre-exposed compared to the non pre-exposed group when haloperidol was injected in shell (t_(17)_ = 2.2, p < 0.05). However, there was no evidence of LI after haloperidol injection in core (t < 1). LI was similarly non-significant after saline injection in the shell. Unexpectedly given the limited number of pre-exposures, saline injection at core coordinates appeared to potentiate LI as there was a clear difference in the level of conditioning to the CS between the pre-exposed and non pre-exposed animals (t_(8)_ = 3.45, p < 0.05).

## 4 Discussion

Experiment 1 tested the effects of 6-OHDA lesions targeted at the core and medial shell of the NAc on LI under experimental conditions (10 pre-exposures) designed to prevent the emergence of LI in vehicle-injected controls. Consistent with previous reports vehicle-injected animals failed to show significant LI under these experimental parameters (e.g., Ruob et al., 1998, Schiller and Weiner, 2005; Nelson et al., 2010a). Similarly, there was no evidence of LI in animals with 6-OHDA lesions targeted at the core. The level of LI is assessed in terms of the difference in the level of suppression between the NPE and PE animals in each drug/placement group (see e.g., Feldon et al., 1991), and on this basis LI was clearly potentiated by infusion of 6-OHDA into the shell. Similarly, in Experiment 2, haloperidol enhanced LI when injected in shell but not core.

In Experiment 1, assay by HPLC revealed differential depletion by infusion site with about 65% depletion in each of the NAc subregions targeted. This magnitude of DA depletion is less than that earlier reported after local injection of 6-OHDA (e.g., Cousins et al., 1993; Salamone et al., 2001; Correa et al., 2002). However, these studies were total NAc depletions and with more selective procedures higher levels of DA depletion produce greater concomitant depletion in the non-targeted subregion (Sokolowski and Salamone 1998; Nelson et al., 2010b, in press). Moreover, 6-OHDA-induced DA depletion in the NAc has been shown to result in differential changes in the morphology of medium spiny neurons within the core and shell (Meredith et al., 1995). Such regionally-specific differences in the response to DA depletion, rather than simply gross changes in DA levels, may contribute to behavioral dissociations of the kind observed here.

In the present study, although infusion of 6-OHDA did nonetheless deplete DA in the NAc region adjacent to the intended site, this depletion was significantly less than found in the target structure (see Table 1b). The recent evidence of widespread intra-accumbal projections (van Dongen et al., 2005) may underlie the pattern of catecholaminergic dennervation that we and others have observed with this kind of lesion (Sokolowski and Salamone 1998; Boye et al., 2001; Sellings and Clarke, 2003; Sellings et al., 2008; Nelson et al., 2010b, in press). The lesions, in particular the core, did also lead to changes in DA levels within the DLS, PL and IL cortex in line with the known pattern of corticostriatal projections of the NAc (Berendse et al., 1992; Groenewegen et al., 1999; Haber et al., 2000). There is no consistent evidence that these comparison regions play any direct role in LI (Solomon and Staton, 1982; Lacroix et al., 2000; but see Ellenbroek et al., 1997). Pretreatment with desipramine provided a degree of neurochemical selectivity but NA was not uniformly protected. A role for NA in LI cannot be precluded in view of the well-established effects of the catecholamine agonist amphetamine on LI. However, the behaviorally-effective shell lesion produced only minimal changes in NAc NA levels, suggesting that the behavioral effects seen at test are meditated by DA depletion within shell NAc.

Taken in isolation, Experiment 1 cannot tell us at what stage of the procedure effects were mediated. In line with the findings of Joseph et al. (2000), Experiment 2 confirmed that LI enhancement was mediated at the conditioning stage of the procedure, consistent with the switching hypothesis of LI. Although the present experiments with few pre-exposures were not standard to test for LI abolition, and do not routinely yield LI in naïve rats, haloperidol and 6-OHDA in core tended to attenuate rather than enhance LI relative to the corresponding core control groups. Indeed in Experiment 2, LI in the saline-core group achieved significance, whereas LI in the haloperidol-core group was not significant. This raises the possibility that intra-core haloperidol injection was not without effect, but rather than potentiating LI as at the shell placement, LI was reduced by haloperidol in the core. However, the unexpected difference in the saline-injected control groups means that this possibility cannot be distinguished from the effect on LI of the saline injection. Thus the effects of haloperidol injection in core should be further examined using non-injected groups to control for any mechanical damage produced by the saline infusion, which based on electrolytic and excitotoxic lesion studies would be expected to enhance LI (Weiner, 2003; Gal et al., 2005; Schiller et al., 2006). Notwithstanding this issue, the present data can be interpreted as showing, that under the specific conditions of this study, that DA depletion and blockade in the core do not potentiate LI.

Together the results of Experiments 1 and 2 underscore the dissociable roles of core and shell subregions of the NAc in mediating the expression of LI and suggest that specifically reduced activity at DA D2-like receptors within the medial shell leads to enhanced LI. This conclusion is supported by *in vivo* studies of DA release in NAc showing that the expression of LI is associated with reduced DA release within the medial shell but not core NAc. Specifically, it has been demonstrated that extracellular levels of DA are increased in the medial shell when a CS is paired with an aversive event but that this conditioned release is eliminated following non-reinforced pre-exposure to the CS (Murphy et al., 2001; Jeanblanc et al., 2002; 2003).

In terms of the switching hypothesis of LI, increased DA efflux in the shell at the time of conditioning may serve to release the switching mechanism, posited to reside in the core, from shell-mediated inhibition and allow the organism to respond adaptively to the CS-reinforcement contingency (Weiner, 2003). As stimulus pre-exposure abolishes this phasic DA response, the core switching mechanism is not activated and hence behavior is controlled by the CS-no event contingency acquired at pre-exposure. In Experiment 1, the 6-OHDA-induced depletion of shell DA could mimic the effects of non-reinforced pre-exposure by further attenuating this conditioned DA release and thus lead to the emergence of LI even under conditions (limited pre-exposure) that do not produce LI in controls. A similar mechanism could underlie the potentiation of LI produced in Experiment 2, where DA D2 receptors were selectively antagonized by haloperidol injection in shell. Within this framework, reduced DA function in the core would not be expected to affect LI as phasic DA release in shell would still activate the core-mediated switching mechanism. This is exactly the pattern of results we observed here with 6-OHDA lesions to, or haloperidol injection in, core NAc. Seen in a broader context of accumbal function, this proposition is consistent with data indicating that the shell modulates the attribution of motivational valence to stimuli, while the core mediates behavioral output to conditioned stimuli (Meredith et al., 2008). Seen in the context of the LI model of schizophrenia, the results of Experiment 2 are consistent with other evidence that NAc shell is the neural locus of action of antipsychotic drugs (Deutch et al., 1992; Dilts et al., 1993).

## Figures and Tables

**Table 1a:** Levels of DA and NA (pmol/μg protein) of vehicle-injected controls, core and shell lesioned animals in core, shell, dorsolateral striatum (DLS), prelimbic (PL) and infralimbic (IL) cortices.

	DA			NA		
	Vehicle	Core lesion	Shell lesion	Vehicle	Core lesion	Shell lesion
Core sample	9.688	3.466	5.439	0.325	0.214	0.285
	(± 1.51)	(± 0.502)	(± 0.563)	(± 0.028)	(± 0.024)	(± 0.028)
Shell sample	5.294	2.945	1.933	0.717	0.464	0.605
	(± 0.742)	(± 0.501)	(± 0.233)	(± 0.091)	(± 0.049)	(± 0.058)
DLS sample	10.594	7.128	8.289	0.173	0.1393	0.1266
	(± 0.583)	(± 0.461)	(± 0.343)	(± 0.114)	(± 0.01)	(± 0.012)
PL sample	0.07	0.052	0.049	0.221	0.179	0.178
	(± 0.008)	(± 0.007)	(± 0.006)	(± 0.016)	(± 0.012)	(± 0.017)
IL sample	0.82	0.039	0.064	0.244	0.165	0.207
	(± 0.011)	(± 0.006)	(± 0.006)	(± 0.024)	(± 0.024)	(± 0.018)

**Table 1b:** Percentage difference in DA and NA levels of core and shell lesioned animals compared to vehicle-injected controls in the 5 brain regions assayed. * significant difference from sham, ^†^ significant difference from other lesion group, p < 0.05, t-test.

	DA		NA	
	Core lesion	Shell lesion	Core lesion	Shell lesion
Core sample	− 64.4%*^†^	− 43.7%*^†^	− 52.3%^†^	− 7.7%
	(± 5.1)	(± 5.8)	(± 10.9)	(± 19.2)
Shell sample	− 46.7%*^†^	− 68.4%*^†^	− 38.8%*	− 9.3%^†^
	(± 9.0)	(± 3.9)	(± 14.0)	(± 18.9)
DLS sample	− 35.5%*^†^	− 16.9%^†^	− 25.6%*	− 24.6%*
	(± 4.2)	(± 3.9)	(± 6.9)	(± 7.4)
PL sample	− 28.8%	− 24.5%	− 17.5%	− 20.8%
	(± 9.5)	(± 9.1)	(± 19.4)	(± 16.9)
IL sample	− 52.5%*^†^	− 13.6%^†^	− 31.6%*	− 20.4%
	(± 6.9)	(± 10.5)	(± 16.1)	(± 17.0)
